# Correlation Between Lung Density Changes Under Different Dose Gradients and Radiation Pneumonitis—Based on an Analysis of Computed Tomography Scans During Esophageal Cancer Radiotherapy

**DOI:** 10.3389/fonc.2021.650764

**Published:** 2021-05-26

**Authors:** Feng Du, Hong Liu, Wei Wang, Yingjie Zhang, Jianbin Li

**Affiliations:** ^1^ School of Medicine, Cheeloo College of Medicine, Shandong University, Jinan, China; ^2^ Department of Radiation Oncology, Zibo Municipal Hospital, Zibo, China; ^3^ Shandong Cancer Hospital, Cheeloo College of Medicine, Shandong University, Jinan, China

**Keywords:** esophageal cancer, radiation therapy, computed tomography value, lung dose, radiation pneumonia

## Abstract

**Purpose:**

To assess the relationship between different doses of radiation and lung density changes and to determine the ability of this correlation to identify esophageal cancer (EC) patients who develop radiation pneumonitis (RP) and the occurrence time of RP.

**Methods:**

A planning computed tomography (CT) scan and a re-planning CT scan were retrospectively collected under institutional review board approval for each of 103 thoracic segment EC patients who underwent radiotherapy (RT). The isodose curve was established on the planning CT with an interval of 5 Gy, which was used as the standard for dividing different gradient doses. Planning CT and re-planning CT scans were matched and the mean lung CT value (HU) between different doses gradients was automatically obtained by the software system. The density change value (ΔHU) was the difference of CT value between each dose gradient before and after treatment. The correlation between ΔHU and the corresponding dose was calculated, as well as the regression coefficients. Additionally the correlation between ΔHU and the occurrence and time of RP (< 4 weeks, 4–12 weeks, > 12 weeks) was calculated.

**Results:**

The radiation dose and ΔHU was positively correlated, but the correlation coefficient and regression coefficient were lower, 0.261 (P <0.001) and 0.127 (P <0.001), respectively. With the increase of radiation dose gradient, ΔHU in RP≥2 group was higher than that in RP<2 group, and there was significant difference between two groups in ΔHU_20-25_, ΔHU_25-30_, ΔHU_30-35_, ΔHU_35-40_, ΔHU_40-45_, ΔHU_45-50_ (p<0.05). The occurrence time of RP was negatively correlated with the degree of ΔHU (P<0.05), with a high correlation coefficient (Y = week actual value −0.521, P < 0.001) (Y = week grade value −0.381, P = 0.004) and regression coefficient (Y = week actual value −0.503, P<0.001) (Y = week rating value −0.401, P=0.002).

**Conclusions:**

A relationship between radiation dose and lung density changes was observed. For most dose intervals, there was an increase of ΔHU with an increased radiation dose, although low correlation coefficient. ΔHU were obvious after irradiation with dose ≥20 Gy which was closely related to the occurrence of RP. For patients with RP, the more obvious ΔHU, the earlier the occurrence of RP, there was a significant negative correlation between them.

## Introduction

Radiation induced lung injury (RILI) is one of the main side effects of radiotherapy (RT) for esophageal cancer (EC). The early manifestation of this injury is radiation pneumonitis (RP), which can occur during RT or within a few months after RT (1). Radiation induced lung tissue reactions, such as inflammatory cell infiltration, exudation, fibrin deposition and fibrosis, can lead to changes in lung density (2). Some studies suggest that computed tomography (CT) density changes not only reflect the changes of radiation-induced lung tissue, but also can be used to quantify radiation-induced lung injury (3–5). At present, some studies have focused on the correlation between the changes of lung density after RT and the dose of lung irradiation (6–9), however, it is still unclear whether there is a correlation between lung radiation dose and lung density changes (ΔHU) during RT and whether ΔHU during RT are related to the occurrence of RP. To this end, this study analyzed the correlation between lung dose-density changes among different dose gradients in patients with EC based on planning CT before RT and re-planning CT during RT, and discussed the feasibility of this correlation predicting RP occurrence and its occurrence time.

## Materials and Methods

### Patient*-S*pecific *C*linical and Treatment Data

A retrospective study of 103 patients with thoracic segment EC treated with RT in our institution from September 2015 to December 2019 was conducted. Patients included in the study were screened from a follow-up of 247 patients undergoing RT for EC. We deleted 144 patients with large difference in lung volume (LV) between planning CT and re-planning CT because the LV was different before and after RT due to the influence of respiratory movement, and the larger LV movement affected the accuracy of image registration. The inclusion criteria were as follows: (1) Karnofsky performance score (KPS) ≥ 70; (2) no previous history of thoracic RT; (3) intensity-modulated radiotherapy (IMRT) and received ≥ 50 Gy RT; and (4) the change of LV between planning CT and re-planning CT was < 8%. As an additional note, we selected and analyzed patients whose LV difference was less than 10% between planning CT and re-planning CT, and found that ΔHU was between 30 to 35 HU, and the error caused by such difference could be ignored in this study. Therefore, we included and excluded patients according to the criteria of less than 10%. We further calculated the LV difference ratio for planning CT and re-planning CT of these patients and found that both of them were less than 8%, so 8% was taken as cutoff. The exclusion criteria were as follows: (1) general pulmonary infection unrelated to RT; and (2) treatment break of more than 7 days during RT. The study was conducted in accordance with the Declaration of Helsinki [as revised in 2013). The study was approved by our institutional (SDTHEC201703014)] and individual consent for this retrospective analysis was waived.

### Patient *C*haracteristics

There were 78 males and 25 females with a median age of 66 years (49–88 years). The histology included squamous cell carcinoma, adenocarcinoma and small cell carcinoma. All RT patients underwent Philips large-diameter CT scan. The median radiation dose was 60 Gy. Most patients separately received induction, concurrent, or sequential chemotherapy based on individualized treatment strategy. The chemotherapy regimens contained cisplatin with fluorouracil (PF regimen), cisplatin with paclitaxel (TP regimen). In all patients, including 19 cases without chemotherapy, 21 cases with induction chemotherapy, 28 cases with induction and concurrent chemoradiotherapy, 25 cases with concurrent chemoradiotherapy, and 10 cases with consolidation chemotherapy after RT. The median chemotherapy cycles were 4 (range, 2–6 cycles). The doses and adjustments of chemotherapy regimens followed the guidelines of the National Comprehensive Cancer Network (NCCN) for EC. The incidence of RP in these patients was as follows: 48 cases of grade 0, 19 cases of grade 1, 29 cases of grade 2, 5 cases of grade 3 and 2 cases of grade 4. Approximately one third/two thirds of patients with/without RP were incidental. (It should be noted that these data cannot represent the incidence of RP in our institution, because we purposely screened these patients to meet the requirements of the experiment.) Further patient characteristics are shown in **Table 1**.

**Table 1:** Patient characteristics.

	n	**%**
**Gender**		
Male	78	75.7
Female	25	24.3
**Age (years)**		
<60	24	23.3
≥60	79	76.7
**Tumor stage**		
II	14	13.6
III	50	48.5
IV	39	37.9
**cN category**		
cN0	18	17.5
cN +	85	82.5
**Tumor location**		
upper thoracic	17	16.5
mid thoracic	18	17.5
lower thoracic/GEJ	68	66.0
**Histology**		
squamous cell carcinoma	96	93.2
adenocarcinoma	2	1.9
small cell carcinoma	5	4.9
**Delivered dose (Gy)**		
<60	27	26.2
≥60	76	73.8
**Chemotherapy mode**		
No	19	18.5
Induce	21	20.4
Concurrent	25	24.2
Induce +concurrent	28	27.2
Consolidate	10	9.7
**Chemotherapy regimen**		
No	19	18.5
TP	26	25.2
PF	58	56.3

PF, cisplatin with fluorouracil; TP, cisplatin with paclitaxel.

### Planning CT and Re-planning CT

Philips large-aperture CT (Phillips Medical Systems, 96 Highland Heights, OH, USA) was used for simulated positioning and helical scanning. The planning CT and re-planning CT were both taken with free breathing. These patients were not treated with 4D average image scanning technique, but with free-breathing conventional enhanced CT images. Physical planning of RT is performed on enhanced CT. Its specific scanning parameters are as follows: Aperture 85 cm, effective scanning aperture 60 cm, voltage (120 kVp), current (260 mA), Pixel matrix size (512×512), field of view (FOV, 50×50 cm), pitch (0.938), slice thickness (3.0 mm). All patients underwent intensive scanning from the cricothyroid membrane to the upper pole of both kidneys. The reset time (re-planning time) was divided into 30 to 30.8 Gy/15 to 17 fraction (22 cases), 39.6 to 40 Gy/20 to 22 fraction (49 cases), and 50 to 50.4 Gy/25 to 28 fraction (32 cases). In the past, most of the RT of EC in our center was done with elective node irradiation (ENI), so re-planning time was performed at a prescribed dose of 50 to 50.4 Gy/25 to 28 fraction, and then the dose was increased to 60 Gy for gross tumor volume (GTV) and regional metastatic lymph node (GTVn). Subsequently, involved-field irradiation (IFI) was adopted, so the re-planning time was performed at the prescribed dose of 39.6 to 40 Gy/20 to 22 fraction. Now, we still adopt IFI, but we choose to re-planning time in the prescription dose to 30 to 30.8 Gy/15 to 17 fraction, the main reason is to consider different esophageal tissue types sensitivity to RT, tumor regression speed is also different (mushroom umbrella and esophageal cavity type is sensitive to radiation therapy), thus to ahead of re-planning time to reduce the volume of GTV, and reduces the toxicity of the lung and heart. Into the group of three different re-planning time of patients was randomly selected, on the one hand, in order to inspect or verify the existence of normal lung tissue of ray high sensitivity (i.e., whether the early low dose radiation rays have an effect on radiation pneumonia), on the other hand was to investigate to accept an equal dose of lung tissue affected by the time the correlation between lung dose density change.

### RT Planning Design and Normal Tissue Constraints

The Eclipse treatment planning system (Varian Medical Systems, Palo Alto, CA, Version 13.5.35) was adopted for the RT planning design for all eligible patients. IMRT was administered by a Varian Linac Accelerator, with 6-MVX ray and 95% planned target volume (PTV), and radiation doses of 50 to 66 Gy (median dose of 60 Gy) and 1.8 to 2.0 Gy/fraction, 5 times/week were prescribed. IMRT adopts fixed field static intensity modulation technique, and five to seven fields of coplanar irradiation fields are uniformly divided according to the specific situation of the case. The required target parameters are then set, and the dose distribution is obtained by inverse calculation of the treatment planning system. The dose distribution was then graded (stratified), and each field was decomposed into a series of subfields. IMRT does not include sIMRT or volumetric modulated arc therapy (VMAT). The target area includes GTV, CT imaging visible esophageal tumor and GTVn. The clinical target volume (CTV) is the upper and lower outward expansion of the esophageal tumor and related lymphatic drainage area. The planned target volume (PTV) has an outward expansion of the CTV of 6 to 8 mm. In addition, the existence of two different radiation modes (ENI and IFI) results in different CTV/PTV sizes.

Normal tissue constraints were prioritized in the following order for treatment planning purposes: maximum spinal cord dose of 45 Gy, relative volume of total lung treated with ≥ 5 Gy (V5) ≤ 60%, relative volume of total lung treated with ≥ 20 Gy (V20) ≤ 28%, MLD ≤ 20 Gy, relative volume of the heart treated with ≥ 30 Gy (V30) ≤ 40%, and relative volume of the heart treated with ≥ 40 Gy (V40) ≤ 30%.

### Establishment of Isodose Curve and Image Registration

**Figure 1:**
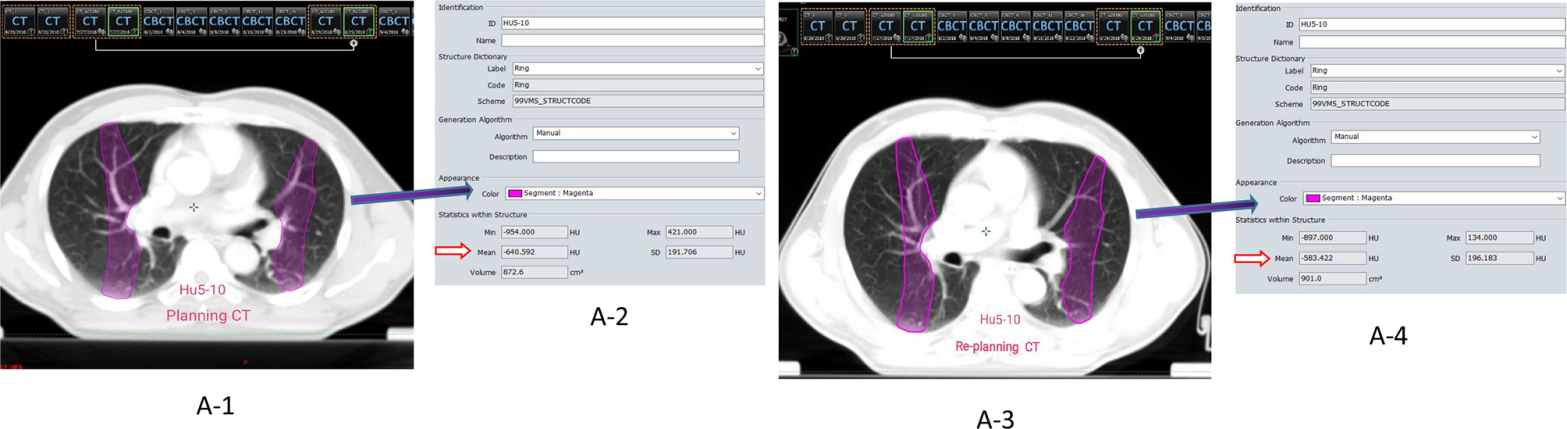
(A_1-4_) The dose gradient interval of HU_5-10_ was selected as an example. ①A-1/A-3 represent the images of the planning CT and the re-planning respectively (Rigid Registration); ②A-2/A-4 represent the parameters of the gradient interval of HU_5-10_, respectively (Mean**HU: mean density value); ③Calculation of density difference (ΔHU) = Planning CT (HU_mean_) − Replanning (HU_mean_).

Firstly, the Eclipse RT planning system was used to establish isodose curve with 5 Gy interval on the enhanced planning CT image, and then the dose interval of different gradient (5, 10, 15…….50 Gy) was established, that is ≤ 5 Gy (HU_0-5_), 5 to 10 Gy (HU_5-10_)…….45 to 50 Gy (HU_45-50_). The system automatically obtained the lung average CT value (HU) of each gradient dose interval. Secondly, using the automatic registration function of the system, the planning CT image is taken as the reference image, the re-planning CT image is taken as the floating image, and the rigid image registration (RIR) is carried out and the local mismatch area is manually adjusted. We used the delineation function of the planning system to adjust the interval outside the lung tissue in the re-planning CT layer by layer into the lung (especially the local mismatch area falling on the mediastinum and chest wall tissue) under the condition of the lung window. Finally, the different dose intervals of the planning CT were copied to the re-planning CT images, and the HU of each dose interval of the re-planning CT were obtained, as shown in **Figure 1A_1-4_**.

The dose maps of the planning CT in this study were scaled according to different re-planning times and then transferred to the re-planning CT. We first set the dose distribution maps of the prescription dose of 30 to 30.8 Gy/15 to 17 fraction, 39.6 to 40 Gy/20 to 22 fraction,and 50 to 50.4 Gy/25 to 28 fraction in the planning CT, instead of the actual prescription dose for the treatment of patients (for example, the prescription dose diagram of 60 Gy or higher), and then transferred the dose distribution maps to the re-planning CT of different times. In short, we have used physical dose maps and not dose maps corrected for fractionation.

### Follow-Up and Evaluation of RP

The follow-up items included chest CT, physical examination and clinical symptoms. Patients were evaluated weekly during RT, and followed up at one month after completion of the initial treatment and then were followed up every 2 to 3 months, at least until half a year after the end of RT. The diagnosis and classification of RP were confirmed by two senior radiologists (professional years ≥5 years) and one radiologist. The RP was graded in accordance with the Common Termination Criteria for Adverse Events, version 4.03 (CTCAE 4.03).

### Statistical Analysis

All statistical analyses were based on SPSS 17.0 (IBM, Armonk, NY, USA). Independent sample t-test or Mann Whitney rank sum test were used to compare the ΔHU between RP≥2 group and RP<2 group at different dose intervals. Spearman correlation analysis was used to analyze the correlation between lung radiation dose and ΔHU, and between ΔHU and the occurrence time of RP. Non constant linear regression model was used for regression analysis. P-values ≤ 0.05 were considered statistically significant.

## Results

### Lung Volume Changes in Planning CT and Re-planning CT

103 patients with EC in thoracic segment were treated with re-planning according to different RT time or radiation dose, including 30 to 30.8 Gy (22 cases), 39.6–40 Gy (49 cases), 50–50.4 Gy (32 cases). The absolute value of lung volume difference between planning CT and re-planning CT was 212.61 ± 142.98 cm^3^, 194.06 ± 131.21 cm^3^, and 174.00 ± 127.77 cm^3^. The percentage of lung volume change was 7.09 ± 5.67%, 6.50 ± 4.84%, and 5.36 ± 3.86%, as shown in **Table 2**.

**Table 2:** Changes of LV parameters in patients with planning and re-planning CT $\left(\bar{x}\pm s\right)$ .

Volume parameter	30–30.8 Gy (n=22)	39.6–40 Gy (n=49)	50–50.4 Gy (n=32)
Planning LV (cm^3^)	3285.38 ± 730.09	3255.27 ± 912.55	3300.80 ± 940.23
Re-planning LV (cm^3^)	3340.92 ± 701.04	3312.07 ± 851.66	3356.63 ± 973.94
Absolute value of LV difference (cm^3^)	212.61 ± 142.98	194.06 ± 131.21	174.00 ± 127.77
Percentage change of LV (%)	7.09 ± 5.67	6.50 ± 4.84	5.36 ± 3.86

LV, lung volume.

### ΔHu Corresponding to Different Dose Gradients Between Planning CT and Re-planning CT

Overall, 65% of the values for ΔHu of the lung parenchyma range between 0 and 200 HU. About 35% of the values were negative, the majority of those between −50 and 0 HU. At the 39.6- to 40-Gy time point a significantly higher HU increase was observed compared to the other time points. **Table 3** shows ΔHU_mean_ with their corresponding 95% confidence interval (CI) for all data.

**Table 3:** ΔHU in different dose gradients of patients in different re-planning groups.

Dose gradient (Gy)	30–30.8 Gy		39.6–40 Gy		50–50.4 Gy	
	ΔHU_mean_	95% CI	ΔHU_mean_	95% CI	ΔHU_mean_	95% CI
HU_0-5_	20.79	1.07–40.52	12.20	−2.84 to 27.24	0.83	−16.66 to 18.32
HU_5-10_	17.01	2.50–31.51	51.47	−75.71 to 178.64	−5.05	−23.85 to 13.76
HU_10-15_	19.68	3.88–35.47	7.42	−0.62 to 20.85	1.93	−17.22 to 21.09
HU_15-20_	21.46	4.78–38.14	3.92	−17.01 to 24.85	10.33	−10.16 to 30.82
HU_20-25_	42.34	23.49–61.19	97.36	−39.01 to 233.74	32.66	6.25–59.06
HU_25-30_	34.34	12.24–56.44	83.89	−52.90 to 220.69	19.48	−4.46 to 43.42
HU_30-35_	–	–	30.50	4.27–56.72	23.15	−3.16 to 49.46
HU_35-40_	–	–	41.30	15.32–67.29	35.56	8.18–62.94
HU_40-45_	–	–	–	–	44.00	16.88–71.11
HU_45-50_	–	–	–	–	44.13	20.30–67.96

ΔHUx, change of lung density between X gradients.

Analysis of all the effective values obtained from 103 patients showed that the correlation coefficient (r) of ΔHu was 0.261, and the regression coefficient (b) was 0.127. The difference was statistically significant (P<0.001). Although there was a correlation between different doses and ΔHU, the correlation was weak. Then the correlation between different doses and ΔHU was analyzed according to the different time of replanning. At 30.0 to 30.8 Gy, the correlation coefficient and regression coefficient of ΔHu in different dose gradient were 0.139 (P = 0.111) and 0.129 (P= 0.103). The difference was not statistically significant (P >0.05), indicating that there was no correlation between dose and ΔHu during this periods. The correlation coefficient and regression coefficient between them were 0.237 (P< 0.001) and 0.049 (P= 0.951) at 39.6–40.0 Gy, and 0.302 (P< 0.001) and 0.118 (P= 0.034) at 50–50.4 Gy. The difference was statistically significant (P<0.001). In these two periods, there was a correlation between dose and ΔHu, and the correlation was better than that of the whole, as shown in **Table 4**. The results showed that ΔHu were correlated with time even under the same dose gradient.

**Table 4:** Correlation and regression between dose intervals and ΔHU.

Groups	N	r	*P-*value	b	*P-*value
All	103	0.261	0.000	0.127	0.000
30–30.8 Gy	22	0.139	0.111	0.129	0.103
39.6–40 Gy	49	0.237	0.000	0.049	0.951
50–50.4 Gy	32	0.302	0.000	0.118	0.034

r, correlation coefficient; b, regression coefficient.

### The Correlation Between ΔHu in Different Dose Intervals

**Figure 2:**
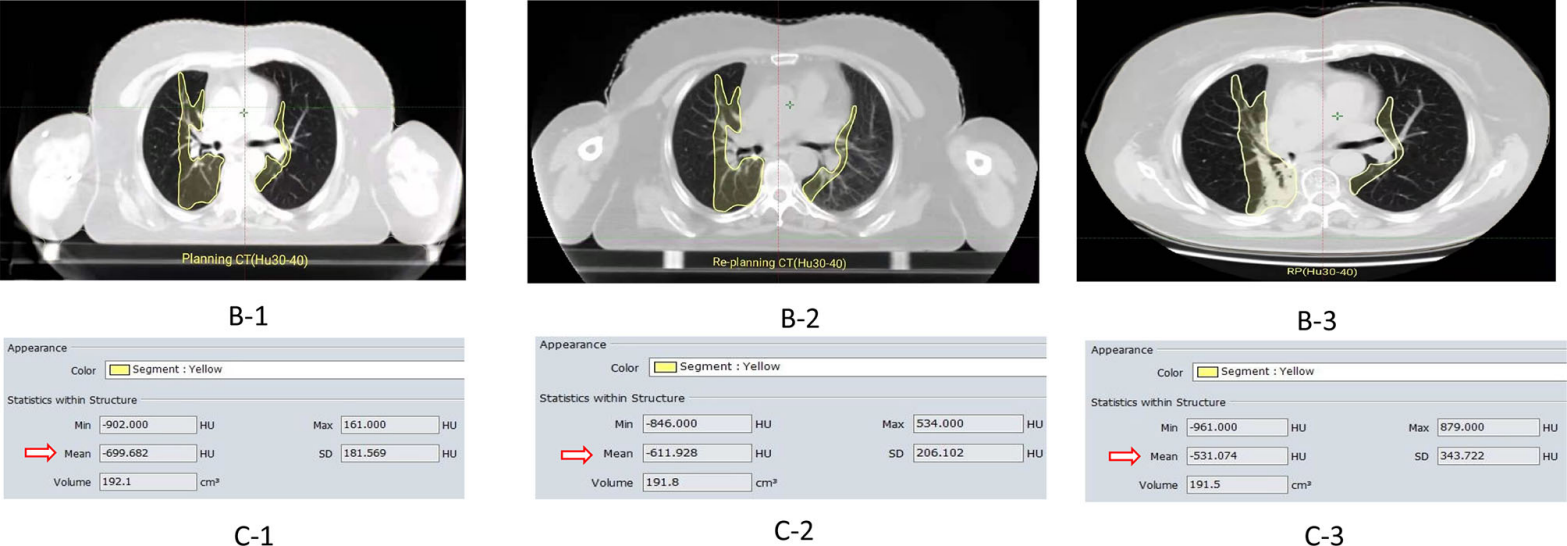
①B-1/-B2 represent the images of the planning CT and the re-planning CT in the HU_30-40_ dose gradient(the yellow curve),respectively. B-3 is the lung image of radiation pneumonia after radiotherapy;②C-1/C-2/C-3 represent CT density values(Hu)in the same or adjacent CT plane in planning CT, re-planning CT, and radiation pneumonia CT respectively (Hu_30-40_); ③ For example, (ΔHU: 87.754)=Planning CT (Hu:-699.682)—Re-planning (Hu:-611.928 Hu).

In the whole group, there were 67 patients (65.05%) RP<2 grade and 36 patients (34.95%) with RP≥2 grade. With the increase of lung radiation dose, ΔHU was more obvious in the RP≥2 group. The RP≥2 group and RP<2 group in ΔHU_20-25_ (51.32 ± 48.70 _VS_ 24.50 ± 50.07), ΔHU_25-30_ (44.37 ± 49.43 _VS_ 8.14 ± 49.06), ΔHU_30-35_ (41.54 ± 47.03 _VS_ 8.49 ± 46.59), ΔHU_35-40_ (48.61 ± 46.91 _VS_ 17.53 ± 53.49) ΔHU_40-45_ (24.12±40.83 VS 8.66±33.59), ΔHU_45_–_50_ (24.32 ± 38.99 _VS_ 7.49 ± 32.62) differences were statistically significant (p<0.05), as shown in **Table 5**. In addition, planning CT, re-planning CT, and post-radiotherapy CT of a patient with grade 2 RP were selected to demonstrate the relationship between this dose-density (ex. HU_30-40_) and high-grade RP, as shown in **Figures 2B_1-3_, C_1-3_**.

**Table 5:** Comparison of the ΔHu in different dose gradients between RP<2 grade and RP≥2 grade.

Dose gradient (Gy)	＜2 grade (n, 67)	≥2 grade (n, 36)	t -value	Z-value	P-value
ΔHU_0-5_	3.95 ± 38.19	12.63 ± 43.20	−1.051	–	0.296
ΔHU_5-10_	−4.25 ± 35.12	40.98 ± 24.68	–	−0.788	0.430
ΔHU_10-15_	0.85 ± 37.63	10.79 ± 37.55	−1.279	–	0.204
ΔHU_15-20_	0.85 ± 42.59	24.05 ± 39.19	−2.709	–	0.108
ΔHU_20-25_	24.50 ± 50.07	51.32 ± 48.70	−2.817	–	0.010
ΔHU_25-30_	8.14 ± 49.06	44.37 ± 49.43	–	−2.926	0.003
ΔHU_30-35_	8.49 ± 46.59	41.54 ± 47.03	–	−3.110	0.002
ΔHU_35-40_	17.53 ± 53.49	48.61 ± 46.91	–	−3.145	0.002
ΔHU_40-45_	8.66 ± 33.59	24.12 ± 40.83	–	−2.220	0.026
ΔHU_45-50_	7.49 ± 32.62	24.32 ± 38.99	–	−2.075	0.038

Δ Hux, lung density change value between X gradients.

### The Correlation Between the Mean Value of ΔHu and the Occurrence Time of RP

**Figure 3:**
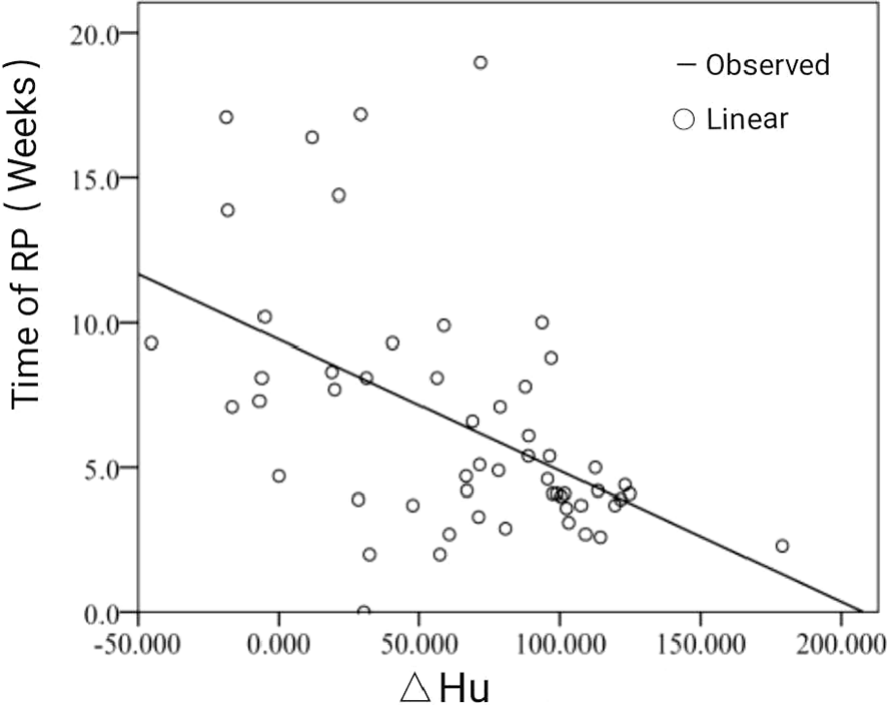
The linear fitting graph of RP occurrence time (y) andΔHu (x).

According to the follow-up results, the occurrence time of RP was divided into three periods: less than 4 weeks (during RT to 4 weeks after RT), 4 to 12 weeks (4–12 weeks after RT), and more than 12 weeks (from 12 weeks after RT to the end of follow-up). The results showed that 18 patients (32.7%) with RP occurred within 4 weeks after RT, 31 patients (56.4%) occurred within 4 to 12 weeks, and only six patients developed RT more than 12 weeks. The actual value and grade value of Y= week correlation coefficient are −0.521 (P<0.001) and −0.381 (P=0.004). The actual value and grade value of Y = week regression coefficient are −0.503 (P<0.001) and −0.401 (P=0.002). Correlation analysis showed that the occurrence time of RP was negatively correlated with ΔHu. This means that a greater change in HU was inversely proportional to the time to RP. The linear fitting graph of RP occurrence time andΔHu, as shown in **Figure 3**.

## Discussion

Radiation pneumonitis (RP) is one of the major complications of thoracic radiation therapy (10, 11). If RP occurs, it will seriously affect the quality of life and survival prognosis (12). The accurate prediction of RP is essential to facilitate individualized radiation dosing and leads to maximized therapeutic gain. The pathological changes of RP are mainly microvascular structure damage, resulting in capillary congestion and thrombosis. After further development of the disease, protein rich fluid exudates into the interstitium and enters the alveoli through the damaged alveolar epithelium, which belongs to aseptic pulmonary inflammation. After the lung tissue received different doses of radiation, the degree of reaction of alveolar tissue is different, this kind of tissue change process can be reflected by the early lung tissue density change, and the early density change is closely related to the occurrence of clinical RP. Therefore, the purpose of our study is to find the characteristics of lung density changes corresponding to different doses of lung irradiation, so as to provide help for early identification of RP and clinical prevention measures.

CT is not only the main means of pre-treatment diagnosis and post-treatment evaluation of pulmonary diseases but also an early and objective measurement tool for RP (13–15). In 1988, some scholars reported (16) that CT scanning was used to quantitatively measure radiation-induced lung disease. Subsequently, the changes of lung CT density and the relationship between lung density and dose effect after RT for breast cancer and lung cancer were also reported successively (17–19). CT quantitative measurement of lung image mainly through the quantitative analysis of density, CT density quantitative analysis unit is Hounsfield unit (HU). Bertelsen et al. (20) found that in cone beam computed tomography (CBCT) images obtained relatively early in the course of fractionated RT, the density change (ΔHu) of lung tissue is related to the local radiation dose, which can be found earlier in the treatment process. Although this ΔHu does not necessarily mean that there will be pulmonary toxicity, RP usually shows an increase in density in healthy lung tissue (21). The biological relationship between radiation, and this tissue reaction has not been fully understood, but the early ΔHu may be related to the local inflammatory response, microvascular endothelial cell damage and vascular leakage caused by RT (22).

In IMRT mode, the dose distribution of lung receiving additional radiation is uneven. It is obviously not accurate to evaluate the lung dose density response relationship by using the whole lung or most of the lungs. Our study is not to analyze the relationship between the change of whole lung Δ Hu and lung dose, but to use the RT planning system to analyze the correlation between the lung volume receiving different gradient radiation and the corresponding lung density change, and this analysis is based on the dynamic analysis of different reset time (re-planning CT) in RT. After analyzing the lung dose-density relationship among different gradients, we tried to establish the curve of the relationship between them. Unfortunately, it was not successful. These results are different from previous studies (6, 7, 23, 24) on the dose-density relationship of lung after RT. They have established a dose-density curve and found that with the increase of radiation dose, lung density increases, and this lung density dose-dependent increase has a time-dependent characteristic. Although we did not establish the curve trend of the two, we found that there was a correlation between the lung-dose density of different gradients in RT (P < 0.001), although the correlation was weak (r= 0.261). This is similar to the study of Schröder et al. (6), which found that there was a statistically significant correlation between the changes of lung density after RT and the radiation dose, but the correlation coefficient was low (r =0.162). However, Bernchou et al. (14) did not find a significant correlation between the changes of lung density and radiation dose based on the analysis of CBCT images in RT.

At present, RP is mainly interpreted by CT images, and ΔHu is a continuous variable that can be objectively measured. Therefore, the analysis of ΔHu during RT may be used to predict the occurrence of RP after thoracic RT. Ma et al. (19) research shows that there was a dose-response relationship between quantitative CT density changes in lung cancer patients during three-dimensional conformal radiotherapy (3DCRT), and CT density before and after treatment could be used as a predictor of lung injury. Palma et al. (25) found that the quantitative analysis of lung density changes is closely related to the clinical RP score, and CT density measurement using deformable registration technology can quantitatively evaluate radiation-related pulmonary toxicity. In the present study, we found that with the increase of lung radiation dose gradient, lung density of RP≥2 group and RP<2 group increased in different ranges, but ΔHu in RP≥2 group was higher than that in RP<2 group, and there were significant differences between two groups in ΔHU_20-25_, ΔHU_25-30_, ΔHu_30-35_, ΔHu_35-40_, ΔHu_40-45_, ΔHu_45-50_ (all P <0.05). This result shows that the change of lung density is not only reflected in the period after RT, but also has clinical significance in the middle and late stage of RT. The density change in this threshold range is related to the occurrence of RP. As Feghali et al. studies (5), ΔHu in peritumoral region was positively correlated with RP grade. Phernambucq et al. (7) found that the threshold dose of lung dose density change was 30 Gy, and there was only slight density change at low dose level. Aoki et al. (26) analyzed that the minimum dose related to the change of CT lung density was 16 to 36 Gy (median dose was 24 Gy), while Boersma et al. (27) normalized the data to obtain the dose-effect relationship of density change, which had little change in the middle and low-dose range. For such results, studies (18) suggest that the increase of CT density in low-dose areas of the lung may be due to the “relative” blood flow systematically redistributing to the less exposed areas, thus moderately increasing the tissue density or due to anatomical changes and/or inaccurate image registration.

In our study, a significantly higher HU increase was observed at the 39.6 to 40 Gy time point compared to the other time points. Obviously, this point is worth analyzing. Regarding the influence of the radiation dose on ΔHU there was an increase in lung density for increasing radiation doses for most dose intervals (6). However, the magnitude or threshold of density increase is inconsistent among various literature reports (7, 26, 27). We believed that the significantly higher CT value of 40 Gy (HU_20-40_) time point might be related to the increased sensitivity of lung tissue from the 4th week of RT. At this time point, the threshold value was 20 to 40 Gy, that is, alveolar exudation was the most obvious in this range. Thereafter, although the radiation dose increased, the alveolar inflammation began to absorb or counteract the exudate, and the CT value increased less dramatically than before. Therefore, it can be considered that time dependence and dose dependence of RT are two necessary factors to obtain the optimal threshold range of CT value change. It is recommended to perform re-planning CT at the time point of 40 Gy, as this point seems to have the greatest predictive value for RP. However, it is very difficult to determine the cutoff value of ΔHU through this retrospective study, because it is affected by many interfering factors (such as basic lung function, whether concurrent chemotherapy, contrast agent use method). We analyzed the dose-density correlation with higher-grade RP and found that the increase of ΔHU in patients with RP was concentrated in the range of 75 to 110 HU, and high or low ΔHU had a low correlation with the occurrence of high grade RP. At the same time, we also found that the relationship was more pronounced in the area of lung tissue receiving 20 Gy extra irradiation.

As mentioned above, early studies have confirmed that changes in lung density obtained by quantitative analysis do not necessarily represent the inevitability of RP, and this density change changes with time after RT (7, 8, 24). Although lung CT imaging is an effective diagnostic tool for RP, it cannot predict the occurrence time of RP in the early stage, which is a difficult problem in clinical practice. We made a separate analysis of the patients with RP, and found that there was a significant negative correlation between the occurrence time of RP and the change range of lung-dose density. This means that a greater change in HU was inversely proportional to the time to RP. That is, the earlier RP occurred, the more significant the correlation of lung-dose density change was. It may be helpful for clinical early judgment of the occurrence time of RP.

The clinical significance of this study is to explore the correlation between lung radiation dose and lung density changes in esophageal cancer RT, and the relationship between this correlation and the occurrence of RP, so as to make early judgment and intervention for RP≥2 grade. These measures include early use of glucocorticoids to reduce alveolar inflammatory exudation or the use of antioxidant acetylcysteine effervescent tablets to reduce inflammatory response. In addition, other measures include reducing the prescription dose of esophageal tumor and avoiding excessive additional radiation to lung tissue. Most importantly, we found that there were significant differences in ΔHu _20-50_ between RP≥2 and RP < 2. Therefore, we should pay more attention to the parameters V_20_-V_50_ of lung high-dose volume in the planning of RT.

This study used raw data sets without any correction and standardized image scanning mode based on pre-treatment planning CT and in-treatment re-planning CT, which overcomes the Hu change caused by the difference of equipment parameters between planned CT and diagnostic CT. However, there are still many challenges in the pre and post analysis of CT scan of RT, including patient’s age, gender, contrast enhancement degree and time, and chemotherapy factors, which can also lead to the increase or decrease of density (18, 28). What’s more, although we used the body membrane fixation device and purposely enrolled the patients whose lung volume change was less than 8%, but the difference between positioning CT and reset CT scanning due to breathing and weight changes still affected the registration accuracy, thus affecting the accurate measurement of lung density, which brought some errors to the experimental study. This study belongs to exploratory research, the focus is to explore the feasibility of a method. At the beginning of the study design, it is not expected to get positive results, because this is just to assess the relationship between different doses of radiation and lung density changes, however, after the found that the correlation between the two, we further analyzed the value of it in terms of predicting the RP. Due to the limited number of cases, the correlation analysis between Δ Hu and RP at a certain time point cannot be realized. In addition, we have found from some studies (7, 24) that Δ Hu after RT is time-correlated with the occurrence of RP, so it seems meaningless to use a single time point to predict the occurrence of RP. It is a little difficult to realize the clinical practice value of this study in this respect, but this research idea is feasible. Therefore, we will further validate our results in a prospective study by performing multiple re-planning CT (including the last re-planning CT after the completion of all prescription doses) in the same large cohort.

## Conclusions

In conclusion, our study demonstrates that there is a certain correlation between lung dose-density of different gradients during RT for EC. For most dose intervals there is an increase of lung density with an increased radiation dose. The dose-density relationship is reflected by a statistically significant, although low correlation coefficient. Due to possible back ground noise a definite statement regarding a threshold for RT induced density changes is difficult. When the lung irradiation dose was ≥ 20 Gy, there was a significant difference between the RP≥2 group and the RP <2 group in the lung dose-density change, which can be used to distinguish the occurrence of ≥2 RP or not. When the ΔHU of RP patients was observed alone, the more obvious the ΔHU, the earlier the occurrence of RP. There was a negative correlation between them, and the correlation coefficient was large.

## Data Availability Statement

The raw data supporting the conclusions of this article will be made available by the authors, without undue reservation.

## Author Contributions

FD and HL were responsible for analyzing data and writing papers. JL designed experiments to guide the writing and revision of papers. WW and ZY directed the writing and revision of papers. All authors contributed to the article and approved the submitted version.

## Funding

The National Key Research Program of China (no. 2016YFC0904700); National Natural Science Foundation of China (No. 81773287); and The Key Research Development Program of Shandong Province (no. 2016GSF201093).

## Conflict of Interest

The authors declare that the research was conducted in the absence of any commercial or financial relationships that could be construed as a potential conflict of interest.
