# Corrigendum: Correlation Between Lung Density Changes Under Different Dose Gradients and Radiation Pneumonitis—Based on an Analysis of Computed Tomography Scans During Esophageal Cancer Radiotherapy

**DOI:** 10.3389/fonc.2021.786217

**Published:** 2021-10-12

**Authors:** Feng Du, Hong Liu, Wei Wang, Yingjie Zhang, Jianbin Li

**Affiliations:** ^1^ School of Medicine, Cheeloo College of Medicine, Shandong University, Jinan, China; ^2^ Department of Radiation Oncology, Zibo Municipal Hospital, Zibo, China; ^3^ Shandong Cancer Hospital, Cheeloo College of Medicine, Shandong University, Jinan, China

**Keywords:** radiation therapy, computed tomography value, lung dose, esophageal cancer, radiation pneumonia

In the published article, there was an error in **affiliation 1**. Instead of **^1^School of Clinical Medicine, Cheeloo College of Medicine, Shandong University, Jinan, China**, it should be **^1^School of Medicine, Cheeloo College of Medicine, Shandong University, Jinan, China**.

There was another error in **affiliation 3**. Instead of **^3^Department of Radiation Oncology, Shandong Cancer Hospital and Institute, Shandong Cancer Hospital Affiliated to Shandong University, Shandong First Medical University and Shandong Academy of Medical Sciences, Jinan China**, it should be **^3^Shandong Cancer Hospital, Cheeloo College of Medicine, Shandong University, Jinan, China**.

The authors apologize for these errors and state that this does not change the scientific conclusions of the article in any way. The original article has been updated.

## Publisher’s Note

All claims expressed in this article are solely those of the authors and do not necessarily represent those of their affiliated organizations, or those of the publisher, the editors and the reviewers. Any product that may be evaluated in this article, or claim that may be made by its manufacturer, is not guaranteed or endorsed by the publisher.

