# Supplementary material for: Correlation Between Lung Density Changes Under Different Dose Gradients and Radiation Pneumonitis—Based on an Analysis of Computed Tomography Scans During Esophageal Cancer Radiotherapy
Source: Front Oncol. 2021 May 26;11:650764. doi: 10.3389/fonc.2021.650764 (PMC8187904; doi:10.3389/fonc.2021.650764)
Supplement: Supplementary file 1 [file DataSheet_1.pdf]

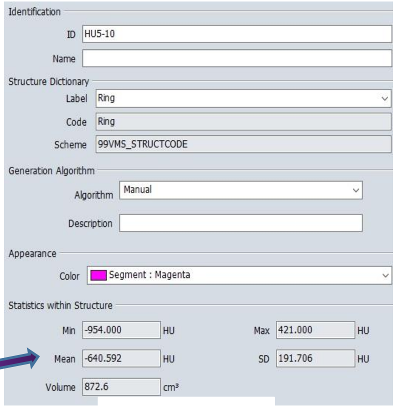

B-1

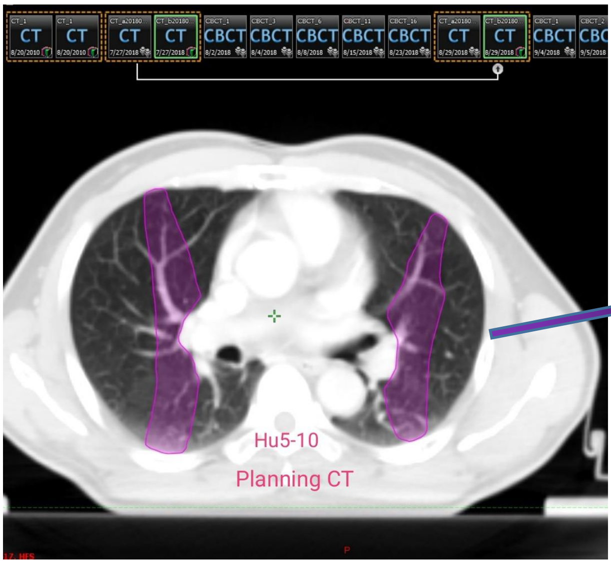

A-1

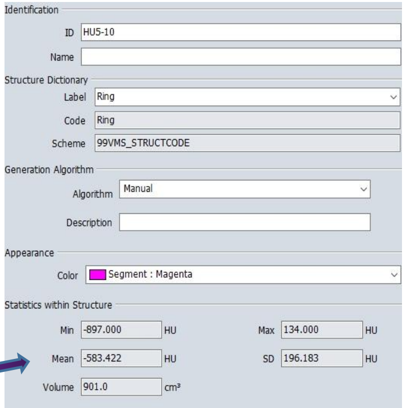

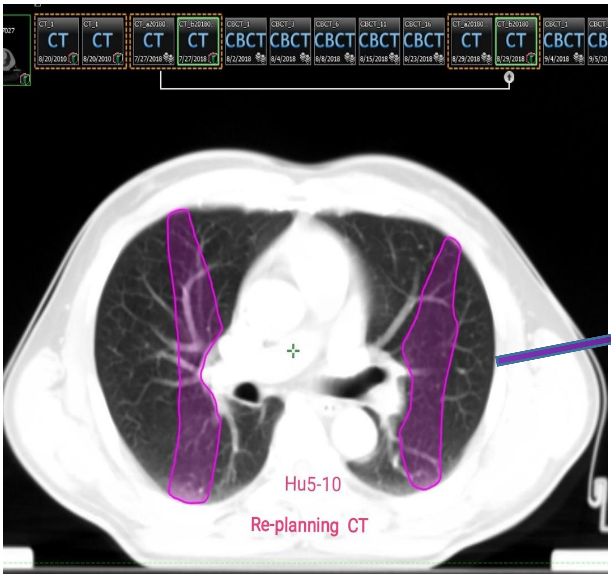

B-2

A-2

In this figure, the dose gradient interval of HU5-10 was selected as an example

① A-1/A-2 represent the images of the planning CT and the re-planning respectively (Rigid Registration)

② B-1/B-2 represent the parameters of the gradient interval of $HU_{5\text{-}10}$, respectively.(Mean**HU: mean density value)

③Calculation of density difference(ΔHU)＝

Planning CT(HU_{mean})-Re-planning(HU_{mean})
