# Supplementary material for: Correlation Between Lung Density Changes Under Different Dose Gradients and Radiation Pneumonitis—Based on an Analysis of Computed Tomography Scans During Esophageal Cancer Radiotherapy
Source: Front Oncol. 2021 May 26;11:650764. doi: 10.3389/fonc.2021.650764 (PMC8187904; doi:10.3389/fonc.2021.650764)
Supplement: Supplementary file 2 [file DataSheet_2.pdf]

①C-1/C-2 represent the images of the planning CT and the re-planning CT in the HU30-40 dose gradient(the yellow curve) ,respectively. C-3 is the lung image of radiation pneumonia after radiotherapy. ②D-1/D-2/D-3 represent CT density values(HU)in the same or adjacent CT plane in planning CT , re-planning CT,and radiation pneumonia CT respectively (HU30-40). ③ E-1/E-2/E3 represent the imaging features of radiation pneumonia (red arrows) on different CT planes.

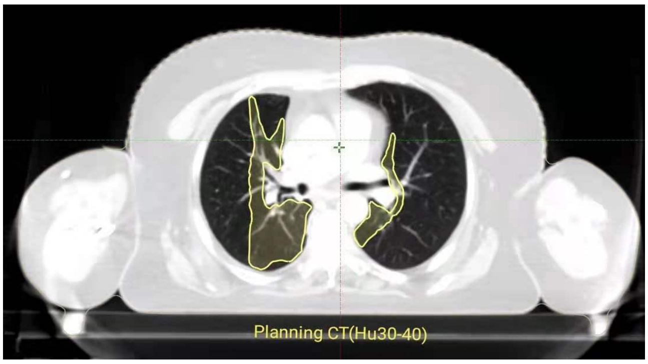

C-1

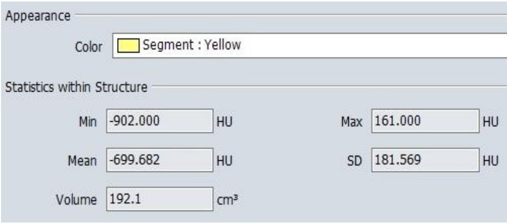

D-1

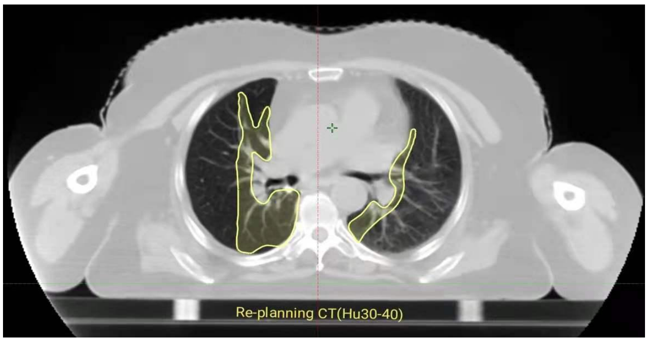

C-2

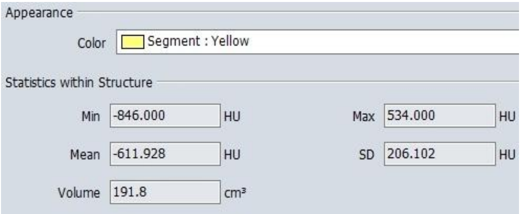

D-2

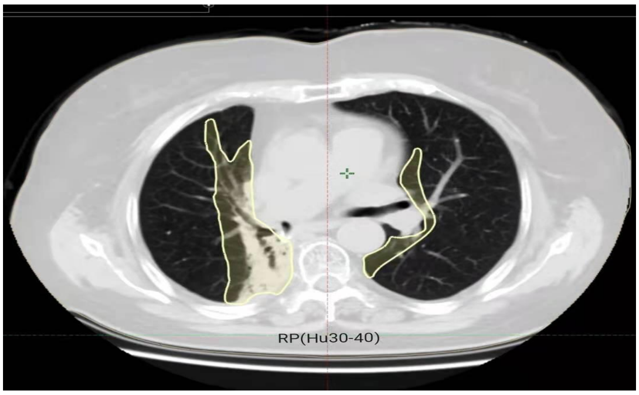

C-3

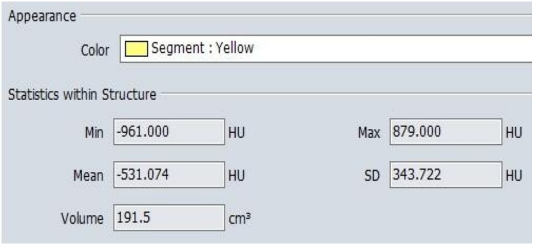

D-3

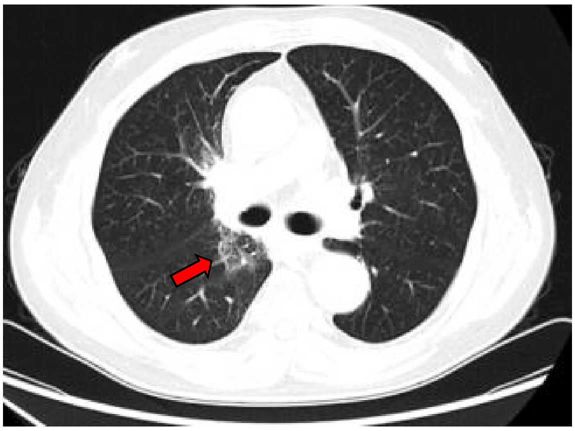

E-1

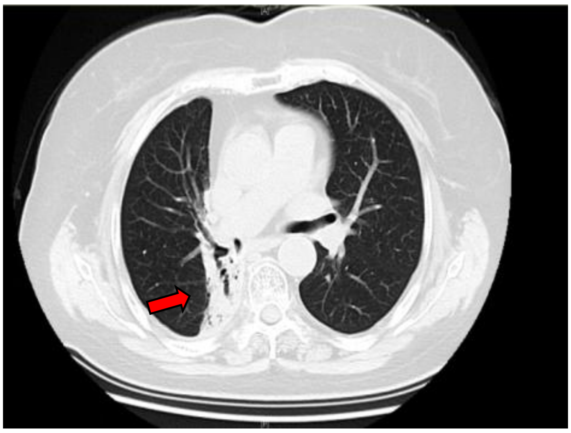

E-2

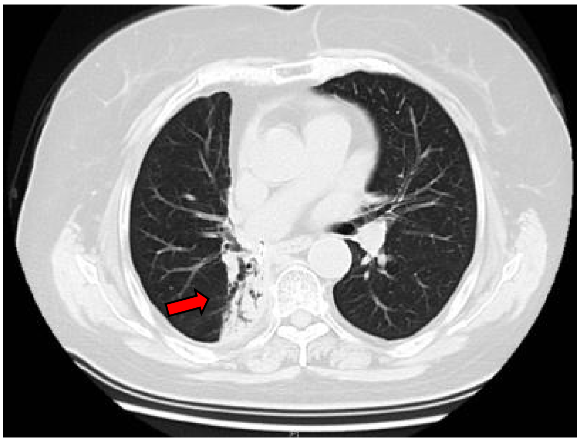

E-3
